# Supplementary material for: The Impact of Different Cultivation Systems on the Content of Selected Secondary Metabolites and Antioxidant Activity of Carlina acaulis Plant Material
Source: Molecules. 2019 Dec 30;25(1):146. doi: 10.3390/molecules25010146 (PMC6983060; doi:10.3390/molecules25010146)
Supplement: Supplementary file 1 [file molecules-25-00146-s001.pdf]

Supplementary material

The impact of different cultivation systems on the content of selected secondary metabolites and antioxidant activity of *Carlina acaulis* plant material

Maciej Strzemski [1,*], Sławomir Dresler [2], Ireneusz Sowa [1], Anna Czubacka [3], Monika Agacka-Mołdoch [3], Bartosz J. Płachno [4], Sebastian Granica [5], Marcin Feldo[6] and Magdalena Wójciak [1,*]

[1] Department of Analytical Chemistry, Medical University of Lublin, Chodźki 4a, 20-093 Lublin, Poland; i.sowa@umlub.pl (I.S.); kosiorma@wp.pl (M.W.)
[2] Department of Plant Physiology and Biophysics, Maria Curie-Skłodowska University, Akademicka 19, 20-033 Lublin, Poland; slawomir.dresler@poczta.umcs.lublin.pl (S.D.)
[3] Department of Plant Breeding and Biotechnology, Institute of Soil Science and Plant Cultivation, State Research Institute, Czartoryskich 8 St., 24-100 Puławy, Poland; annacz@iung.pulawy.pl (A.CZ.); magacka@iung.pulawy.pl (M.AM.)
[4] Department of Plant Cytology and Embryology, Institute of Botany, Faculty of Biology, Jagiellonian University, Gronostajowa 9 St. 30-387 Cracow, Poland; bartosz.plachno@uj.edu.pl (B.P.)
[5] Department of Pharmacognosy and Molecular Basis of Phytotherapy, Faculty of Pharmacy, Medical University of Warsaw, Banacha 1 St., 02-097 Warsaw, Poland; sgranica@gmail.com (S.G.)
[6] Department of Vascular Surgery, Medical University of Lublin, Staszica 11 St. 20-081 Lublin, Poland; marcin.feldo@umlub.pl (M.F.)
* Correspondence: maciej.strzemski@poczta.onet.pl (M.S.); kosiorma@wp.pl (M.W.)

Table S1. Organogenesis on explants originated from *Carlina acaulis* L. plants.

Organ	Medium	Plant morphology	Percentage of explants regenerating into:	
			Roots	Shoots
Roots	A	glabrous	1.06	6.38
		tomentose	0	0.59
	B	glabrous	0	7.32
		tomentose	3.70	10.37
	C	glabrous	0	0
		tomentose	0	0.67
	D	glabrous	0	0
		tomentose	0	0
	E	glabrous	0	0
		tomentose	0	0
Leaves	A	glabrous	3.75	0
		tomentose	0.88	0
	B	glabrous	0	0
		tomentose	0	0
	C	glabrous	0	0
		tomentose	0	0
	D	glabrous	0	0
		tomentose	0	0
	E	glabrous	0	0
		tomentose	0	0

Table 2. Physical characteristics of callus tissue formed on *Carlina acaulis* L. explants.

Medium	Plant morphology	Characteristics of callus formed on explants:	
		Leaves	Roots
A	glabrous	compact, hard, grainy, light green	compact, hard, grainy, cream / beige / light green
	tomentose	compact, hard, grainy, light green / cream	compact, hard, grainy, cream / light green
B	glabrous	friable, grainy, moist, cream	friable, grainy, moist, cream / beige
	tomentose	friable, grainy, moist, cream	friable, grainy, moist, cream / beige
C	glabrous	hard, grainy,moist, cream	hard, grainy, moist, cream / beige
	tomentose	hard, grainy,moist, cream	hard, grainy, moist, cream / light green
D	glabrous	hard, grainy, moist, cream	soft, grainy, moist, cream / beige
	tomentose	hard, grainy, moist, cream	soft, grainy, moist, cream / light green
E	glabrous	soft, grainy, moist, cream / light green	soft, grainy, moist, cream / light green
	tomentose	soft, grainy, moist, cream	soft, grainy, moist, cream / beige

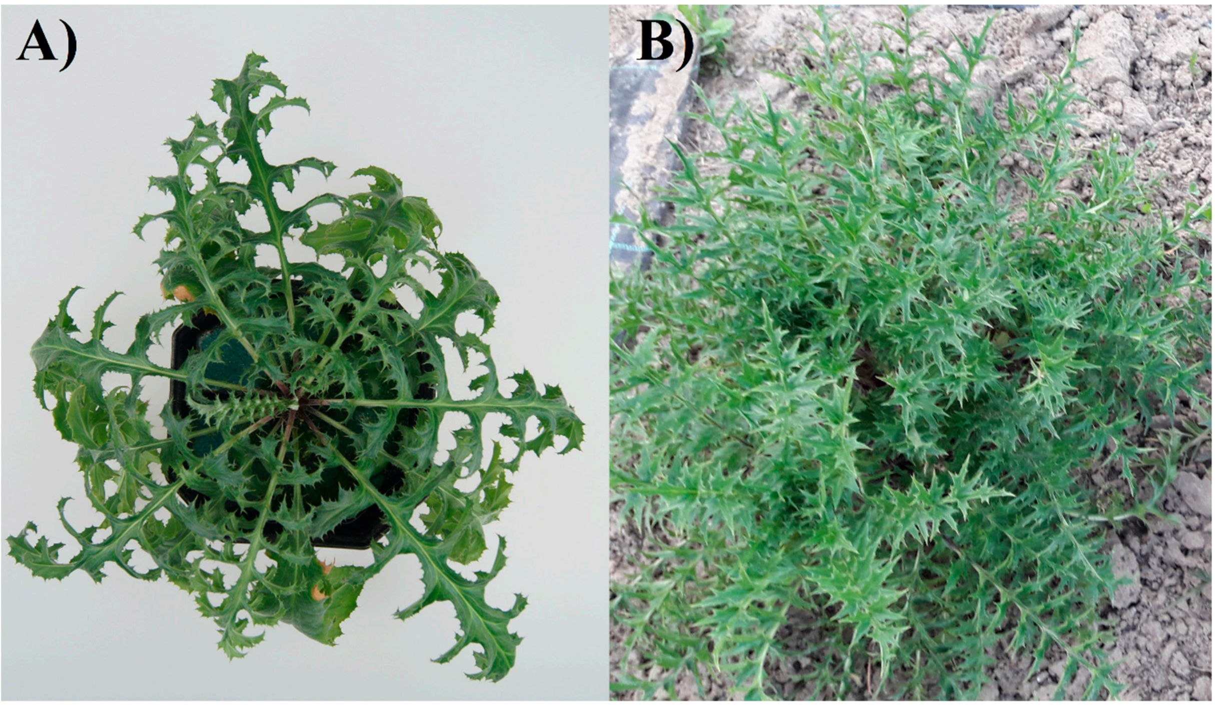

Figure S1. Photographs of examples of *Carlina acaulis* L. plants obtained in hydroponic cultures (A) and soil cultivation (B).

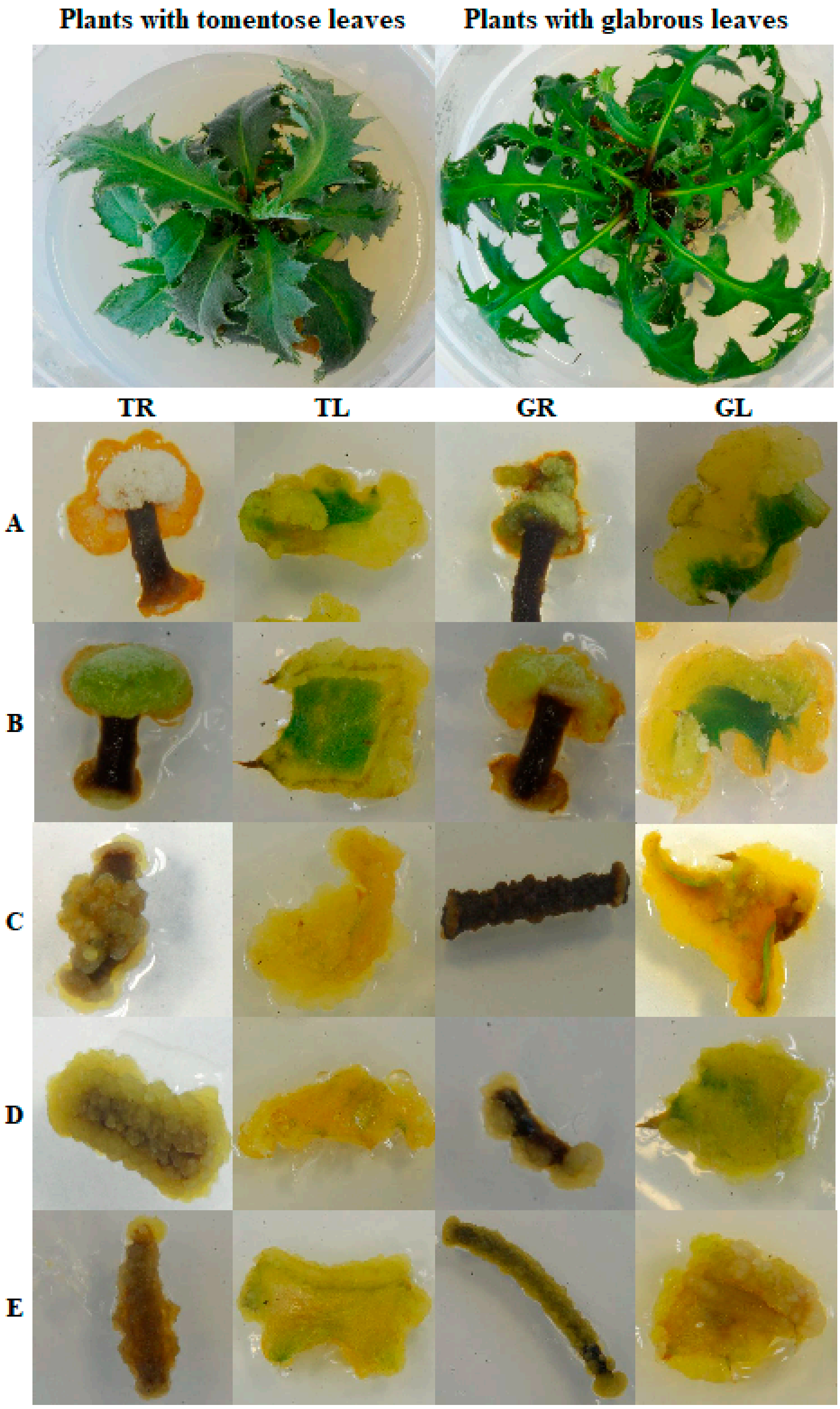

Figure S2. Photographs of sample callus tissues obtained from the roots and leaves of *Carlina acaulis* L. plants, using A-E media.
